# 

**Published:** 1855-11

**Authors:** 


### EDITORIAL.

*Notice.*—We again repeat the request, that all letters and correspondence in relation to subscriptions or other business of the *Journal* should be addressed *exclusively* to "N. S. DAVIS, Chicago, Ill." This same direction was given the commencement of the present volume, and repeated in the *Journal* several times since, and yet we find some subscribers addressing the former Publishers, and afterwards complaining that their letters or remittances are not acknowledged. We hope this notice will be sufficient to correct the evil.

RECEIPTS FROM SEPT. 1st., TO NOV. 8th.

| | | |
|---|---|---|
| | ILLINOIS. | |
| Dr. | J. H. Hollister, | $1,00 |
| " | G. M. Fox, | 2,00 |
| " | C. C. Wearne, | 2,00 |
| " | J. C. Paterson, | 2,00 |
| " | W. R. Griswold, | 1,00 |
| " | S. W. Noble, | 4,00 |
| " | J. B. Paul, | 1,00 |
| " | J. Vandike, | 2,00 |
| " | N. Holton, | 5,00 |
| " | J. Rice, | 2,00 |
| " | J. K. Finley, | 2,00 |
| " | Calvin Wheeler, | 2,00 |
| " | L. Clark, | 2,00 |
| " | Reimbold, | 2,00 |
| " | T. M. Crombie, | 2,00 |
| " | Woodruff, | 2,00 |
| " | J. H. Williamson, | 2,00 |
| " | Allport, | 2,00 |
| " | M. Wiley, | 2,00 |
| " | R. C. Edgerton, | 5,00 |
| " | F. R. Payne, | 2,00 |
| " | J. M. Allen, | 2,00 |
| " | E. Friend, | 2,00 |
| " | R. F. Bennett, | 4,00 |
| " | J. S. Whitmire, | 2,00 |
| " | John Walker, | 2,00 |
| " | W. F. Coleman, | 5,00 |
| " | W. Brenton, | 2,00 |
| " | J. S. Evans, | 2,00 |
| " | A. M. Miller, | 2,00 |
| " | N. B. Butler, | 2,00 |
| " | Albert Atherton, | 2,00 |
| | MICHIGAN. | |
| Dr. | A. Foster, | $5,00 |
| " | S. H. Whittlesey, | 2,00 |
| " | L. B. Coats, | 2,00 |
| | INDIANA. | |
| Dr. | A. Chapman, | $2,00 |
| " | J. H. Moore, | 2,00 |
| " | J. W. Hall, | 2,00 |
| " | D. Proctor, | 2,00 |
| " | Rhea & McConnell, | 2,00 |
| " | T. Newkirk, | 2,00 |
| " | Henry Wehmer, | 2,00 |
| | WISCONSIN. | |
| Dr. | J. C. Mills, | $2,00 |
| " | C. Marsh, | 2,00 |
| " | A. C. Gibson, | 2,00 |
| " | J. B. Dousman, | 2,00 |
| " | Storrs Hall, | 2,00 |
| | IOWA. | |
| Dr. | L. A. Babcock, | $2,00 |
| " | A. E. Smith, | 2,00 |
| " | B. F. Dewey, | 1,00 |
| | CALIFORNIA. | |
| Dr. | Edward Hopkins, | $2,00 |

